# Ultrasound guided placement of the distal catheter in paediatric ventriculoatrial shunts—an appraisal of efficacy and complications

**DOI:** 10.1007/s00381-016-3120-4

**Published:** 2016-05-20

**Authors:** David J. Clark, Aabir Chakraborty, Derek J. Roebuck, Dominic N. P. Thompson

**Affiliations:** Department of Paediatric Neurosurgery, Great Ormond Street Hospital for Children NHS Foundation Trust, Great Ormond Street, London, UK; Department of Interventional Radiology, Great Ormond Street Hospital for Children NHS Foundation Trust, London, UK

**Keywords:** Ventriculoatrial, CSF shunt, Hydrocephalus, Cerebrospinal fluid, Paediatric

## Abstract

**Purpose:**

Ventriculoatrial (VA) shunts are commonly used as a second-line treatment of hydrocephalus when the peritoneum is an unsuitable site for the distal catheter. Many centres now utilise ultrasound and interventional radiology techniques to aid placement of the distal catheter. The purpose of this study was to conduct a contemporary audit of VA shunting in children using interventional radiology techniques for placement of the distal catheter.

**Methods:**

A retrospective analysis of all patients who had VA shunts inserted between June 2000 and June 2010 was conducted using a prospectively updated surgical database and case notes review.

**Results:**

Ninety-four VA shunts were inserted in 38 patients. Thirty-seven patients had been treated initially with ventriculoperitoneal (VP) shunts. Twenty-two patients required at least 1 shunt revision (58 %). The 6-month, 1- and 2-year shunt survival rates were 53, 43 and 27 %, respectively. Blockage was the commonest reason for shunt failure (68 %). The site of failure was proximal (ventricular catheter +/− valve) in 32 % and distal (atrial catheter) in 21 % of cases. The overall infection rate was 6 % per procedure and 11 % per patient. There were 7 deaths, of which 3 were shunt related.

**Conclusions:**

VA shunting provides a viable second-line option for shunt placement in complex hydrocephalus. The causes of shunt failure (blockage, infection and equipment failure) are similar to VP shunting though shunt survival rates are inferior to VP shunts. Ultrasound guided VA shunt placement provides a relatively safe, second-line alternative to the placement of a ventriculoperitoneal shunt when this route is unsuitable.

## Introduction

Despite the current enthusiasm for endoscopic third ventriculostomy, ventricular shunting procedures continue to be the mainstay of hydrocephalus management in children and ventriculoperitoneal shunting remains the preferred first-line option. However, approximately 50 % of ventriculoperitoneal (VP) shunts inserted fail within 2 years [9, 19, 21], with this figure rising to 85 % after 15 years [22]. The majority of VP shunts fail due to obstruction, followed by mechanical failures (migration and disconnection) and infection [22]. Distal shunt failure may occur as a result of adhesions, intraperitoneal infection, ascites and cerebrospinal fluid (CSF) pseudocysts [11, 20]; in these situations, an alternative site for the distal catheter may have to be considered.

Ventriculoatrial shunting (VA) was originally described by Nulsen and Spitz in 1951 and was the prevailing technique of hydrocephalus management for many years [15]. In recent decades, VP shunting has become the preferred treatment since surgical placement is more straightforward and as VA shunts can cause serious complications including pulmonary embolism, pulmonary hypertension [17] and shunt nephritis [1].

Insertion of the atrial catheter was traditionally done through open dissection of the neck to locate a suitable tributary of the internal jugular vein (typically the common facial vein) to cannulate from whence the catheter could be directed to the right atrium. This operation could be particularly challenging in the case of small infants and in cases of revision. Ashker et al. first described a method for percutaneously inserting the distal catheter in 1981 [2]. Since then, this less invasive method has gained widespread acceptance but only a few studies have examined its effect on outcomes in children [5, 6]. We present our experience of VA shunting with percutaneous insertion of the distal catheter under ultrasound guidance as a second-line treatment for childhood hydrocephalus over a 10-year period.

## Materials and methods

This retrospective study was conducted at the Department of Neurosurgery at Great Ormond Street Hospital for Children in London. Patients who had their first VA shunt inserted between the 1st of June 2000 and the 1st of June 2010 were identified from the unit’s prospectively updated operative database. The medical records for these patients were studied as was the operating theatre log. The aetiology of hydrocephalus, age of initial intervention, age at insertion of the first VP shunt, the number of VP shunt revisions prior to VA shunt insertion and the time from first VP shunt insertion to VA shunt insertion as well as the main reasons for failure of intraperitoneal placement were documented for all patients. Surgical time for VA shunt insertion was also noted as was the vein of entry for the atrial catheter.

Outcomes from VA shunt placement were then recorded including perioperative morbidity and mortality, and any infective complications and responsible organisms involved. The time to first VA shunt revision was calculated, and the reasons for shunt failure were also described.

### Technique of VA shunt placement

The procedure of VA shunt insertion used in this series was similar to the technique described by Ashker et al. [2]. The procedure is performed in conjunction with interventional radiology. Having located the internal jugular vein by ultrasound, a transverse incision is made just superior to the clavicle overlying the vein. Under ultrasound guidance, the internal jugular vein is punctured with a 21-gauge needle and a floppy-tipped 0.018-in guide wire is inserted under fluoroscopic control. In small children, the guide wire is advanced through the right atrium to the inferior vena cava to allow insertion of dilators over the stiff part of the guide wire. In larger children, the guide wire need only be advanced into the right atrium. The 0.018-in guide wire is exchanged for a 0.035-in wire, using a coaxial micropuncture introducer system (MAK mini access kit, Merit Medical Systems, Jordan, UT, USA), and a 9-Fr peel-away sheath (Cook Incorporated, Bloomington, IN, USA) is inserted, after serial dilation if this is needed. The distance from the neck incision to the mid-atrium is then measured using intraoperative fluoroscopy and a silastic atrial C-type shunt catheter is cut to this length. The shunt catheter is then fed over the guide wire and passed along the peel-away catheter. The guide wire and peel-away catheter are then removed leaving the shunt catheter in situ which is then attached to the proximal shunt system via a straight connector. A final radiograph is taken on-table to identify the location of the tip of the atrial catheter.

## Results

### Baseline characteristics

Forty-one patients had primary VA shunts inserted during the study period; 3 were excluded due to insufficient data being available. The children in this study had undergone a mean of 4.3 (SD +/− 4.6) VP shunt revisions prior to VA shunt placement (range 0 to 13). The mean age of children at the time of initial VP shunt placement was 1 year (range <1 day to 10.1 years). A total of 94 VA shunts were inserted in 38 patients, an average of 2.5 shunt operations per patient. Twenty-three (61 %) of the patients were male. Post-haemorrhagic hydrocephalus (39 %) and myelomeningocele (13 %) were the most common causes of hydrocephalus; congenital hydrocephalus or cases without clear aetiology comprised 24 % (Table 1).

**Table 1:** Aetiologies of hydrocephalus

Aetiology of hydrocephalus	*n* (%)
Post-haemorrhagic	15 (39)
Hydrocephalus of unknown aetiology	9 (24)
Myelomeningocele	5 (13)
Tumour	3 (8)
Dandy Walker malformation	2 (5)
Post-meningitic	2 (5)
Benign intracranial hypertension	1 (3)
Crouzon’s syndrome	1 (3)

The causes of peritoneal catheter failure included CSF ascites (42 %) which commonly occurred in the context of repeated abdominal surgeries and a prior history intrabdominal sepsis (24 %). Adhesions precluding placement of a peritoneal catheter and pseudocyst formation occurred in 13 and 16 % of cases, respectively (Table 2).

**Table 2:** Reasons for intraperitoneal catheter failure

Reason for choosing atrial route	*n* (%)
Ascites	16 (42)
Infection	9 (24)
Pseudocyst	6 (16)
Insertion difficulties	5 (13)
Unknown	2 (5)

### Perioperative details

**Fig. 1:**
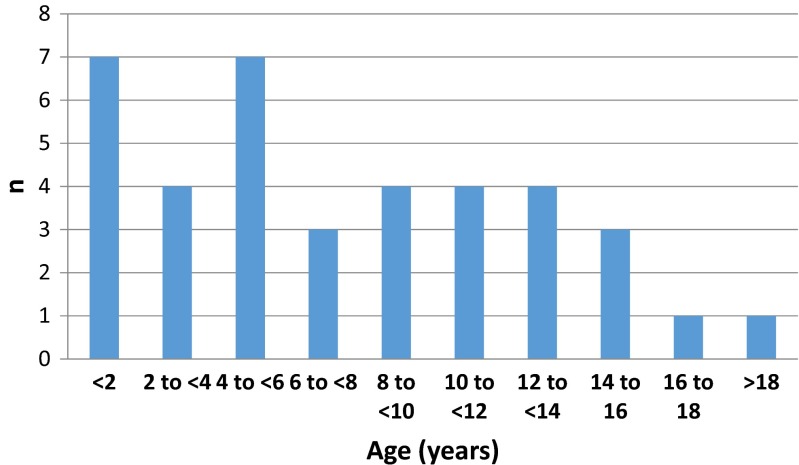
Range of ages at which the primary VA shunt was inserted

Mean age at placement of first VA shunt was 7.6 years (range 0.4 to 19.8—see Fig. 1). The right internal jugular vein was used for access in the majority of cases (55 %); see Table 3 for the frequency of other routes. Major perioperative complications comprised one intraoperative death (1 %) in which a septic embolus dislodged from the infected distal end of a shunt causing a fatal pulmonary embolism. With regard to morbidity, a single patient with a congenital diaphragmatic hernia and mediastinal distortion received an atrial perforation during insertion of the distal catheter resulting in a haemopericardium. Following urgent cardiac surgical repair, she recovered fully without further incident.

**Table 3:** Vein selected for cannulation

Vein used for distal catheter insertion	*n* (%)
Right internal jugular vein	22 (58)
Left internal jugular vein	9 (24)
Right brachiocephalic vein	2 (5)
Left brachiocephalic vein	1 (3)
Unnamed collateral vein in right side of neck	1 (3)
Unknown	6 (16)

### Postoperative course

**Fig. 2:**
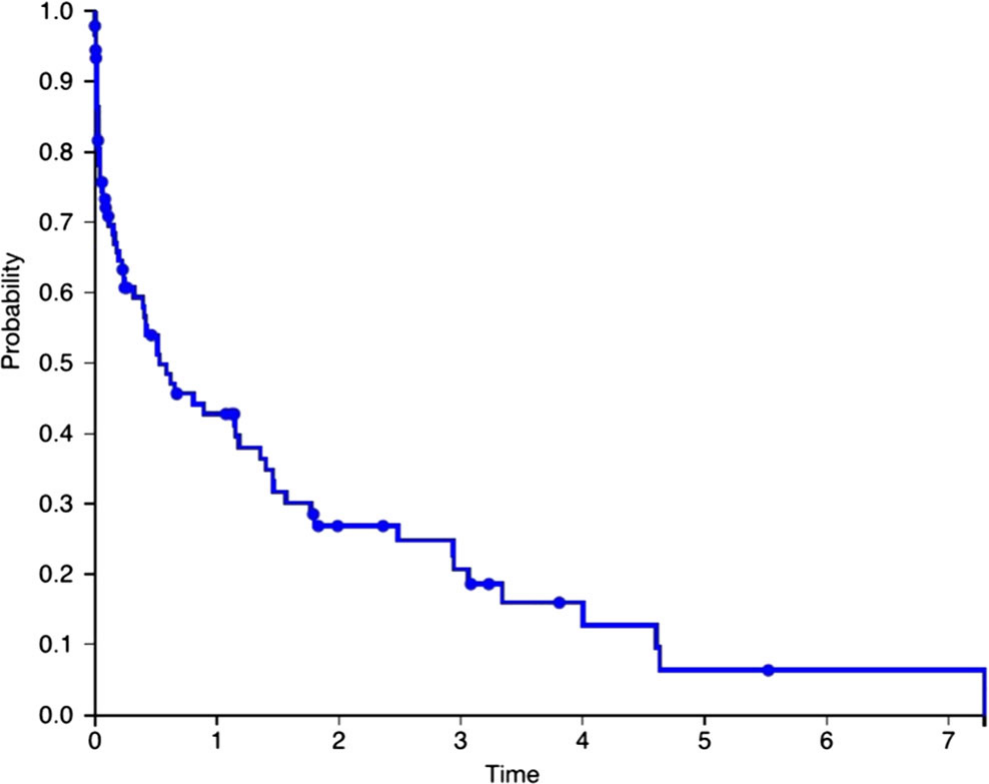
Kaplan-Meier curve of shunt survival over time (in years)

Twenty-two patients required at least one shunt revision (58 %). Of the 94 VA shunts inserted, 33 had not failed at last follow-up (35.1 %). The mean number of revisions following VA shunt insertion was 1.5 (range 0 to 9). The 6-month, 1-, 2- and 3-year shunt survival rates were 53, 43, 27 and 19 %, respectively. Figure 2 shows the Kaplan-Meier graph of shunt survival for all the VA shunts inserted. Mean follow up time was 2.5 (+/− 2.84) years.

**Fig. 3:**
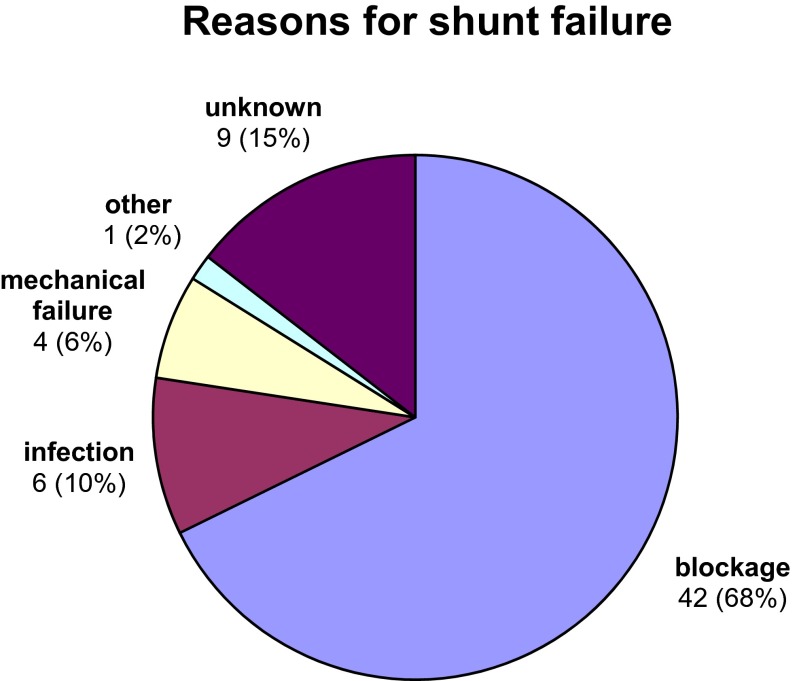
Reasons for shunt failure

The most common reason for revision was shunt blockage (68 %), followed by infection (10 %); see Fig. 2. There were four (6 %) cases of mechanical failure, two (3 %) disconnections and two (3 %) cases of migration of the distal catheter. There was one case of a child whose shunt was externalised due to a suspicious mass on the atrial catheter being identified by echocardiography, but ultimately no organism was cultured from the blood or CSF. Out of 38 primary VA shunts inserted, four shunts became infected (11 %); out of 94 VA shunts inserted, six shunts became infected (6 %). In total, four patients (11 %) had shunt infections. Table 4 summarises the findings on microscopy and culture of cerebrospinal fluid collected. The frequency which each component of the shunt was revised following failure is shown in Table 5. The most commonly revised component was the ventricular catheter (21 %). Proximal (ventricular catheter and/or valve) dysfunction only was the cause of shunt failure in 30 cases (32 %), distal catheter dysfunction only in 20 cases (21 %) and there were nine (10 %) cases where it was unclear which end of the system was responsible for shunt failure (Fig. 3).

**Table 4:** Cerebrospinal fluid microscopy and culture results

Shunt	Microscopy	Culture
1	Gram negative rods	*Enterobacter cloacae*
2	Gram positive cocci	Coagulase-negative staphylococci
3	Gram positive cocci	*Staphylococcus aureus*
4	Not seen	1. *Corynebacterium diphtheria* 2. *Bacillus* sp.
5	Not seen	*Propionibacterium acnes*
6	Not available	Not available

**Table 5:** Revision procedure undertaken following shunt failure

Revision procedure undertaken following shunt failure	*n* (%)
Ventricular catheter revised	20 (21)
Valve revised	6 (7)
Atrial catheter revised	4 (4)
Ventricular catheter and valve revised	4 (4)
Valve and atrial catheter revised	5 (6)
Ventricular and atrial catheter revised	1 (1)
Whole shunt revised	2 (2)
Externalised	12 (13)
Converted to VP	4 (4)
Shunt removed and contralateral EVD placed	1 (1)
Unknown	2 (2)
Shunt had not failed at last follow-up	33 (35)

There were seven (18 %) deaths in this cohort at last follow up; the causes of death are summarised in Table 6. Three (8 %) deaths were related to the patient’s shunt. There was a case of a septic embolus dislodging intraoperatively from an infected VA shunt in a 7-year-old female causing a fatal pulmonary embolism (discussed previously). A 12-year-old male had a cardiorespiratory arrest as a result of blockage of his external ventricular drain 1 day following externalisation. Finally, there was a case of an 8-year-old female with acute renal failure associated with gentamicin, which had been commenced to treat coagulase-negative staphylococcal meningitis.

**Table 6:** Causes of death

Patient	Time from first VA shunt insertion to death	Cause of death
1	2 months, 24 days	Intraoperative septic embolus
2	9 months, 3 days	High grade glioma of posterior fossa with leptomeningeal spread at diagnosis
3	6 months, 9 days	Data unavailable
4	4 months, 21 days	Cardiac arrest
5	2 years, 5 months, 25 days	Data unavailable
6	2 days	Cardiac arrest following shunt blockage
7	7 years, 9 months, 10 days	Acute renal failure secondary to gentamicin toxicity

## Discussion

When the peritoneum becomes unsuitable as a site for the distal catheter in cerebral shunting, opinions vary as to what constitutes the most appropriate second-line management. Whilst VA and ventriculopleural are the most commonly considered alternatives in such a circumstance, it is always prudent to at least consider the option of endoscopic third ventriculostomy. Whilst the perceived likelihood of success might be low, the risk of failure might be more acceptable when considered in the context of VA and ventriculopleural shunts. Our preference has been to use VA rather than ventriculopleural shunting as second-line management once the peritoneal route has failed and ETV has been excluded as a treatment option. The right atrium of the heart is an established alternative site for the distal catheter and VA shunts were the preferred management option for childhood hydrocephalus prior to the introduction of VP shunts. We report the largest series of children receiving percutaneously inserted VA shunts published in the literature to date.

### Patient demographics

The patients receiving VA shunts in this study had all (with one exception) previously had VP shunts which had failed. In a recently published series of 64 patients with VP shunts with a minimum of 15 years follow-up, the mean number of shunt revisions per patient following VP shunt insertion was 2.66 [22]. In our study, the mean number of VP shunt revisions prior to VA shunt insertion was 4.3, suggesting that this cohort likely represents a particularly complex sub-group of shunted patients who by virtue of requiring VA shunting have declared themselves as prone to shunt failure and complications. The majority of our patients had multiple serious comorbidities, many present since birth (including several cases of necrotising enterocolitis of prematurity). The reasons for peritoneal failure are typical and reflect the experience from previous studies [11, 20]. Post-haemorrhagic hydrocephalus and myelomeningocele together constituted over half the cases, and these are both conditions in which there is not infrequently a history of abdominal surgery for other reasons, such as necrotising enterocolitis and urological reconstruction procedures.

### Operative technique

Our technique is similar to that described by Ashker et al.; however, assistance from interventional radiology is sought to identify and cannulate a suitable vein using ultrasound and confirm distal catheter position using fluoroscopy. In terms of the route used for venous access, the internal jugular vein and subclavian vein [5] have previously been used for percutaneous placement of the distal catheter; shunts were inserted into both of these veins in this cohort, and we also report two cases of successful use of the right brachiocephalic vein.

### Shunt failure

Our study is the only large case series of percutaneously inserted VA shunts used as second-line management for childhood hydrocephalus with long-term follow-up published to date. We are aware of one other such series [5] which consisted of only seven patients who were followed up for between 2 and 12 months. In our cohort, shunt survival at 2 years was 27 %. In comparison, level 1 evidence from prospective, randomised controlled trials (Shunt Design Trial, Hakim-Medos Investigator Group) puts shunt survival at 52 % 2 years following VP shunting [9, 19]. If we compare our results to the era in which VA shunts were routinely used as first-line management for childhood hydrocephalus, Fernell et al. reported a shunt survival rate of approximately 40 % at 2 years in a large retrospective case series of VA shunts inserted as first-line treatment for childhood hydrocephalus at two institutions between 1967 and 1983 [7]. As our study was investigating the use of VA shunting as an alternative treatment only when first-line management had failed, this higher rate is to be expected. Dysfunction of the proximal end of the shunt (ventricular catheter and/or valve) was more common in our series than dysfunction of the distal catheter, which is consistent with what is found in VP shunting [21, 22] and suggests this disparity in shunt survival is a manifestation of the complexity of this cohort’s hydrocephalus rather than a reflection of the inadequacy of the atrium as a site for the distal catheter. The majority of shunt failures were caused by obstruction, infection and mechanical failure, which is again similar to VP shunting. It has been observed in the past that shunt failure typically takes place in the first year following VP shunt insertion and failure rates then plateau [22]; in comparison, our data suggests that VA shunts continue to fail at a steady rate even after the first year and, as such, long-term surveillance of these patients should be considered.

### Shunt infections

Eleven percent of patients sustained a shunt infection. In previously reported series, the infection rate per patient in VA shunts inserted through open neck dissection as first-line management for childhood hydrocephalus has been reported as between 4.2 and 11.3 % [7, 14, 24]. One child in our cohort died of gentamicin-associated acute kidney injury following a shunt infection. We observed no cases of endocarditis or shunt nephritis.

### Cardiovascular complications

Pulmonary hypertension is an important complication of VA shunts that we also did not observe. We observed a single case of intraoperative pulmonary embolus, described below. A literature review published in 1993 found that post-mortem diagnoses of pulmonary embolism and pulmonary hypertension were made in 59.7 and 6.3 % of patients with VA shunts, respectively, but a diagnosis was made during life in only 0.4 and 0.3 % of cases, respectively, suggesting these complications are under-recognised by clinicians [17]. Median time between shunt placement and the diagnosis of pulmonary hypertension has previously been reported as 16.5 years [10], which is why we may not have identified any cases in our study.

### Mortality

Seven (18 %) of the children in this series died (including one child with a high grade glioma), though in only three cases (8 %) was death related to the shunt. In a similar study to ours, Tuli et al. retrospectively collected data on all cerebral shunts inserted over a 10-year period at their institution and observed a mortality rate of 13.7 % in the 907 children that presented [23]. This higher mortality rate may be accounted for by the fact the patients selected for inclusion in our study had already had an extensive history of failed VP shunts and, as such, were more likely to have a poorer prognosis compared to a standard population of patients with hydrocephalus managed with shunting. Moreover, the mean age of VA shunt placement in our study was 7.6 years; conversely, five of the seven children who died were under 1 year of age at the time of their death. As they required a VA shunt in infancy, it is again likely their prognosis even prior to shunt insertion was unfavourable.

### Intraoperative adverse events

There was a single intraoperative death in this cohort of 94 VA shunt insertions (1 %). This was due to the dislodgement of a septic embolus during externalisation of an infected shunt resulting in fatal pulmonary embolism. As such, we recommend cautious manipulation of the distal shunt system during revision procedures due to the possibility of embolisation of both infective and non-infective material. In previously published studies, operative mortality pertaining to the open technique ranged from 0 to 2.9 % [16, 24]. In addition, there was a case of atrial perforation in a child with a congenital cardiac anomaly. Taken together, this represents a serious complication rate of 2 %. We believe that, in experienced hands, the percutaneous technique is superior in terms of risk and efficacy when compared with open neck dissection.

### Alternatives to VA shunting

Other than the peritoneum and atrium, the pleura is also often used as a site for the distal catheter of a cerebral shunt [12, 18]. The pleural route has been less popular than the heart because of the risk of pleural effusion and tension hydrothorax [3]. However, the incidence of pleural effusion can be reduced significantly through the use of new technology valves [12]. The gallbladder has recently been explored as a possible viable alternative, and a recent review found these shunts exhibit an overall long-term survival of over 60 % [8]. Novel methods of continuing to use the peritoneum despite previous difficulty have also been conceived, such as laparoscopically assisted insertion [4, 11] and placement of the catheter in the omental bursa [13]; four (11 %) of the patients who had VA shunts inserted during the 10-year period studied at our institution had them replaced by VP shunts at last follow-up. The preferred choice of site for the distal catheter if the peritoneum fails varies between surgeons with sparse evidence to indicate which route is safest or most efficacious. Randomised controlled trials or prospective cohort studies comparing sites are needed to guide future practice.

### Limitations

This study is limited by its retrospective methodology. Moreover, the short follow up time of 2.5 years is a major disadvantage as the serious cardiac and pulmonary complications of this procedure discussed earlier often occur only years after insertion. Shunt complications were only recorded if they resulted in a revision procedure; therefore, data on important non-operatively managed complications (including cases of slit ventricle syndrome) has not been included. Our small sample size makes it difficult to draw any definite conclusions from this study, but it is still the largest series of VA shunts inserted in children using the modern technique of percutaneous insertion under ultrasound guidance reported in the literature to date and as such provides data on shunt survival and adverse outcomes against which future audits of second-line hydrocephalus treatments can be compared.

## Conclusion

VA shunts are an effective second-line intervention for children with hydrocephalus in whom the peritoneum is no longer a suitable site for the distal catheter. The percutaneous ultrasound guided technique is safe with a serious adverse event rate of 2 %. However, patients who require second-line shunt treatments likely represent a sub-group of hydrocephalus patients at particular risk for ongoing shunt-related morbidity and patients and their carers need to be aware of this. Patients with VA shunts require close monitoring for shunt infections and their associated sequelae, as well as for cardiac and pulmonary complications. Moreover, shunt failure rates for VA shunt in this series are high compared with VP shunts. Proximal catheter failure remains the commonest cause of malfunction suggesting that it is the underlying complex hydrocephalus in our cohort rather than the atrial site of drainage that was primarily responsible for failure. We believe that the technique of percutaneous ultrasound guided distal placement justifiably will continue to have a place in the repertoire of complex hydrocephalus management.

## References

[1] Arze RS, Rashid H, Morley R, Ward MK, Kerr DN (1983). Shunt nephritis: report of two cases and review of the literature. Clin Nephrol.

[2] Ashker K, Fox JL (1981). Percutaneous technique for insertion of an atrial catheter for CSF shunting. Technical Note J Neurosurg.

[3] Beach C, Manthey DE (1998). Tension hydrothorax due to ventriculopleural shunting. J Emerg Med.

[4] Bhasin RR, Chen MK, Pincus DW (2007). Salvaging the “lost peritoneum” after ventriculoatrial shunt failures. Childs Nerv Syst.

[5] Britz GW, Avellino AM, Schaller R, Loeser JD (1998). Percutaneous placement of ventriculoatrial shunts in the pediatric population. Pediatr Neurosurg.

[6] Ellegaard L, Mogensen S, Juhler M (2007). Ultrasound-guided percutaneous placement of ventriculoatrial shunts. Childs Nerv Syst.

[7] Fernell E, von Wendt L, Serlo W, Heikkinen E, Andersson H (1985). Ventriculoatrial or ventriculoperitoneal shunts in the treatment of hydrocephalus in children?. Z Kinderchir.

[8] Girotti ME, Singh RR, Rodgers BM (2009). The ventriculo-gallbladder shunt in the treatment of refractory hydrocephalus: a review of the current literature. Am Surg.

[9] Kestle J, Drake J, Milner R, Sainte-Rose C, Cinalli G, Boop F, Piatt J, Haines S, Schiff S, Cochrane D, Steinbok P, MacNeil N (2000). Long-term follow-up data from the shunt design trial. Pediatr Neurosurg.

[10] Kluge S, Baumann HJ, Regelsberger J, Kehler U, Koziej B, Klose H, Greinert U, Kreymann G, Meyer A (2009). Development of pulmonary hypertension in adults after ventriculoatrial shunt implantation. Respiration.

[11] Martin K, Baird R, Farmer JP, Emil S, Laberge JM, Shaw K, Puligandla P (2011). The use of laparoscopy in ventriculoperitoneal shunt revisions. J Pediatr Surg.

[12] Martínez-Lage JF, Torres J, Campillo H, Sanchez-del-Rincón I, Bueno F, Zambudio G, Poza M (2000). Ventriculopleural shunting with new technology valves. Childs Nerv Syst.

[13] Matushita H, Cardeal D, Pinto FC, Plese JP, de Miranda JS (2008). The ventriculoomental bursa shunt. Childs Nerv Syst.

[14] Mazza C, Pasqualin A, Da Pian R (1980). Results of treatment with ventriculoatrial and ventriculoperitoneal shunt in infantile nontumoral hydrocephalus. Childs Brain.

[15] Nulsen FE, Spitz EB (1951) Treatment of hydrocephalus by direct shunt from ventricle to jugular vain. Surg Forum:399–403 14931257

[16] Olsen L, Frykberg T (1983). Complications in the treatment of hydrocephalus in children. A comparison of ventriculoatrial and ventriculoperitoneal shunts in a 20-year material. Acta Paediatr Scand.

[17] Pascual JM, Prakash UB (1993). Development of pulmonary hypertension after placement of a ventriculoatrial shunt. Mayo Clin Proc.

[18] Piatt JH (1994). How effective are ventriculopleural shunts?. Pediatr Neurosurg.

[19] Pollack IF, Albright AL, Adelson PD (1999). A randomized, controlled study of a programmable shunt valve versus a conventional valve for patients with hydrocephalus. Hakim-Medos Investig Group Neurosurg.

[20] Popa F, Grigorean VT, Onose G, Popescu M, Strambu V, Sandu AM (2009). Laparoscopic treatment of abdominal complications following ventriculoperitoneal shunt. J Med Life.

[21] Shannon CN, Acakpo-Satchivi L, Kirby RS, Franklin FA, Wellons JC (2012). Ventriculoperitoneal shunt failure: an institutional review of 2-year survival rates. Childs Nerv Syst.

[22] Stone JJ, Walker CT, Jacobson M, Phillips V, Silberstein HJ (2013). Revision rate of pediatric ventriculoperitoneal shunts after 15 years. J Neurosurg Pediatr.

[23] Tuli S, Tuli J, Drake J, Spears J (2004). Predictors of death in pediatric patients requiring cerebrospinal fluid shunts. J Neurosurg.

[24] Vernet O, Campiche R, de Tribolet N (1993). Long-term results after ventriculoatrial shunting in children. Childs Nerv Syst.

